# Fuzzy Leaky Bucket System for Intelligent Management of Consumer Electricity Elastic Load in Smart Grids

**DOI:** 10.3389/frai.2020.00001

**Published:** 2020-01-31

**Authors:** Miltiadis Alamaniotis

**Affiliations:** Applied Artificial Intelligence Laboratory, Department of Electrical and Computer Engineering, University of Texas at San Antonio, San Antonio, TX, United States

**Keywords:** fuzzy logic, leaky bucket, intelligent management, smart grids, elastic load

## Abstract

This paper frames itself in an informational rich smart electricity grid where consumers have access to various streams of information and make decisions over their daily consumption pattern. In particular, a new intelligent management system to accommodate possible optimal decisions for elastic load consumption is discussed. The energy management system implements a fuzzy driven leaky bucket that manages the elastic load of a consumer by controlling the token rate buffer via a set of four fuzzy variables (among them the electricity price). The goal of this innovative system is to allow loads that are identified as elastic to be scheduled only when it is potentially beneficial to the consumer. To that end, a fuzzy algorithm comprised of a set of rules is developed to manage the token rate of the leaky bucket and through that the decisions over the fate of elastic loads. The developed system is applied on a set of real-world electricity consumption data taken from a residential consumer, and benchmarked against a full scheduling method, where the elastic load is fully scheduled offline. Results exhibit that the proposed fuzzy logic method outperforms the full scheduling method in the vast majority of the cases, i.e., over 79% of the cases with respect to consumption cost. Furthermore, they validate its ability to conduct real time decision making with no human in the loop.

## Introduction

Efficient energy management is a topic of paramount significant toward realization of a smart electricity grid. Given the limitations in the delivery and distribution of electricity imposed by the physical infrastructure of the grid, the issue of intelligent energy management has been revisited (Tsoukalas and Gao, 2008). Advances in information technologies and their coupling with the power system has given rise to the term of smart grid, which is visualized as a resilient infrastructure that secures a 24/7 energy delivery and accommodates the utilization of intermittent sources, such as renewables (Shafiullah et al., 2010). Therefore, smart grids consist of information rich environments where the grid participants have access to a wide variety of heterogeneous modals of information. Consumers, which consist of the vast majority of the grid participants, utilize the available information aiming at satisfying their load demand, with the lowest possible cost (Alamaniotis et al., 2018).

One of the cornerstones upon which the electrical smart grid will be built is the utilization of intelligent systems for the efficient management of energy consumption. Therefore, the energy management from the consumer side should be considered as serving two purposes: (i) the first is morphing the demand in such a way to secure the non-stop grid operation, and (ii) the second is morphing the demand in such a way that the consumer has an economical benefit (i.e., low utility bill) (Alamaniotis et al., 2014). Regarding the consumer, the demand management mainly refers to the scheduling of the operation of the electrical appliances that reside within the premises of the resident. Each electric appliance serves a different purpose, and hence, it has different operational characteristics and different load values. However, the loads in a resident can be assigned to one of the two main classes: (i) inelastic load demand, and (ii) elastic load demand (Thimmapuram and Kim, 2013). The first class (i.e., inelastic demand) encompasses all that load demand that cannot be curtailed or postponed; this type of load has to be satisfied in its entirety at specific time intervals (Alamaniotis et al., 2018). For instance, the electricity demand pertained to fridge operation is inelastic given that the fridge has to operate non-stop to preserve its contents. The second class encompasses all those loads that can be either curtailed or postponed for a later time; in other words, elastic demand may not be satisfied, implying that there is no operation of the respective appliances. An example of elastic load is the coffeemaker, whose operation may take place later or canceled (i.e., consume another drink, e.g., apple juice).

It is evident from the aforementioned types of load demand that the elastic load may be managed, and if efficient, then it may be of benefit to consumer at first, and to the operation of electric grid at second. It is common that the consumer perceives as efficient management the satisfaction of his/her demand at its entirety while keeping the consumption expenses (i.e., electric bill) low (Alamaniotis et al., 2018). Furthermore, if the cost becomes high, then consumers prefer to curtail their demand—and as a result not to fully satisfy their total demand (Babar et al., 2013). Therefore, management of elastic load becomes a complex decision making that is affected by several factors. Such an environment requires that the decision making is conducted in an automated way given that the human consumer cannot afford making decisions 24/7. The factor that has the most weight in decision making pertained to load consumption is the electricity prices. A dynamically varying electricity price has given rise to the price directed electricity markets, where prices are driven by econometric models to encourage or discourage the electricity consumption (Saez-Gallego et al., 2016). In such markets, consumers must follow the prices 24/7 and make respective purchase decisions that reduce the expenses (Chrysikou et al., 2015). Though the idea sounds appealing, it is impossible for humans to non-stop monitor the market prices and decide accordingly. Therefore, automated systems that make decisions on behalf of the human consumer are needed (Jin and Mechehoul, 2010).

In this manuscript, the automated management of consumer's elastic load demand in the context of smart grids is of interest. Several methods that fall under the general approach of “demand response” have been proposed to deal with elastic load management. The majority of those systems adopt various modeling and analytical approaches, encompassing methods from machine learning and statistics. In particular, in Yang et al. (2016) a multi-objective stochastic optimization formulation is used for modeling the relation between prices elasticity and loads, while in McKenna and Keane (2014) a Monte Carlo simulation approach for probabilistic modeling of resident's elastic consumption is discussed. Furthermore, a hybrid method that integrates artificial neural networks with wavelets is introduced in Paterakis et al. (2016), and a method for clustering the consumer's profiles based on their elastic demand is introduced in Dasgupta et al. (2019). In Vázquez et al. (2011) the management system aims at improving the elasticity of loads in smart homes by using pervasive systems and statistical information taken from loads in the country of Austria. In addition, a logarithmic modeling for direct elastic load control is proposed in Farahani et al. (2012), while a model predictive driven utility function for elastic loads control is presented in Shi et al. (2018). A faithful method that performs management of distributed energy resources by using a pricing scheme is introduced in Mhanna et al. (2017), and a dynamic price scheme are the focal point in Althaher et al. (2015) where control of the operation of smart appliance takes into consideration the comfort zone of the consumer. In Hubert and Grijalva (2012) and Tsui and Chan (2012) the appliance control in the context of demand response is modeled as complex optimization whose solution is the optimal in terms of cost for the home energy consumption management, while in Erdinc et al. (2014) an optimization problem for household consumption that takes into consideration electric vehicle demand is presented. Furthermore, a demand response tool of home appliances in the event of renewable energy availability is presented in Althaher and Mutale (2012), and an algorithmic approach that takes into consideration the rebound effect in demand response at home consumption is presented in Li et al. (2012). An intelligent DR method that integrated home load forecasting and predetermines an operational schedule of appliance based on the time of use electricity prices is introduced in Ozturk et al. (2013), with a similar approach focusing on high power consumption appliance is proposed in Pipattanasomporn et al. (2012). A different approach where the consumers report their power usage and allow the utility to decide about the appliance operation is discussed in Cao et al. (2012), while in Kamyab et al. (2015) the DR problem is considered as a game between multiple suppliers and customers without delving into the appliance level.

Notably, there is a high amount of works that have focused on forecasting as the way to perform load management. The vast majority of these works are based on artificial intelligence tools aiming at accurately predicting the load demand as discussed in Di Santo et al. (2018) and Fallah et al. (2018). The aforementioned works do not directly deal with the management of the elastic load of the consumer and they do not assume smart appliances that handle only elastic loads, or they do it only as fully correlated with prices. Furthermore, the use of artificial intelligence tools for elastic load is very limited, allowing room for new and more sophisticated methods. In addition, none of the current works fully exploit the connectivity and the informational rich environments of smart grids. Therefore, there is need for new intelligent methods that manage the elastic loads of a consumer who is connected to the smart grid.

In this paper a new intelligent system for managing the elastic load of a consumer is presented. The proposed system implements a fuzzy leaky bucket (Aeron, 2010) as the mean to manage the elastic load of the electricity consumer. In particular, the proposed system is based on the development of a fuzzy system that utilizes several pieces of information (Tsoukalas and Uhrig, 1997), including electricity prices, to control the token rate of the leaky bucket. The *contribution* of the paper contains: (i) the development of a fuzzy system that implements a leaky bucket approach, (ii) the application of the fuzzy leaky bucket for elastic load management, (iii) the utilization of several variables beyond electricity prices for decision making, and (iv) the introduction of a fully autonomous energy management system, and the (v) introduction and testing of a real time decision making system in smart grids. The concept of fuzzy leaky bucket has been previously in controlling of power flow in power system nodes in Alamaniotis et al. (2016), while a simple implementation for appliance scheduling in smart homes in Alamaniotis and Ktistakis (2018). Thus, the objectives of this paper are: (a) present in full details the fuzzy leaky bucket as elastic load home management, (b) test the proposed method in real world data, and (c) highlight the conclusions made by methodology testing.

The roadmap of the paper is as follows. In the next section, a brief description of the leaky bucket approach utilized for control is given, while in section Fuzzy Leaky Bucket Elastic Load Management System the developed management system is introduced and its steps are explained. Section Testing Results on Elastic Load Management presents the application of the system in a set of real world-data consumption files and discusses the results. At last, section Conclusion concludes the paper and summarizes the main points of it.

## Background: Leaky Bucket Approach

The leaky bucket is an algorithmic approach that has found wide use in several engineering applications where control of a process is requested. It mimics the ways that a water filled bucket which has a leak, may overflown when the incoming flow rate is higher than the leakage rate. In general, it has been used for controlling the frequency of discrete events or to limit the actions associated with those events. The most illustrative application of leaky bucket is in the domain of communication networks (packet switched computer networks) where it is used for controlling the data packet transmission by defining the channel bandwidth (Bertsekas et al., 1992). Overall, the leaky bucket approach ensures that the rate of some sequence of discrete events remains within strictly defined limits.

**Figure 1:**
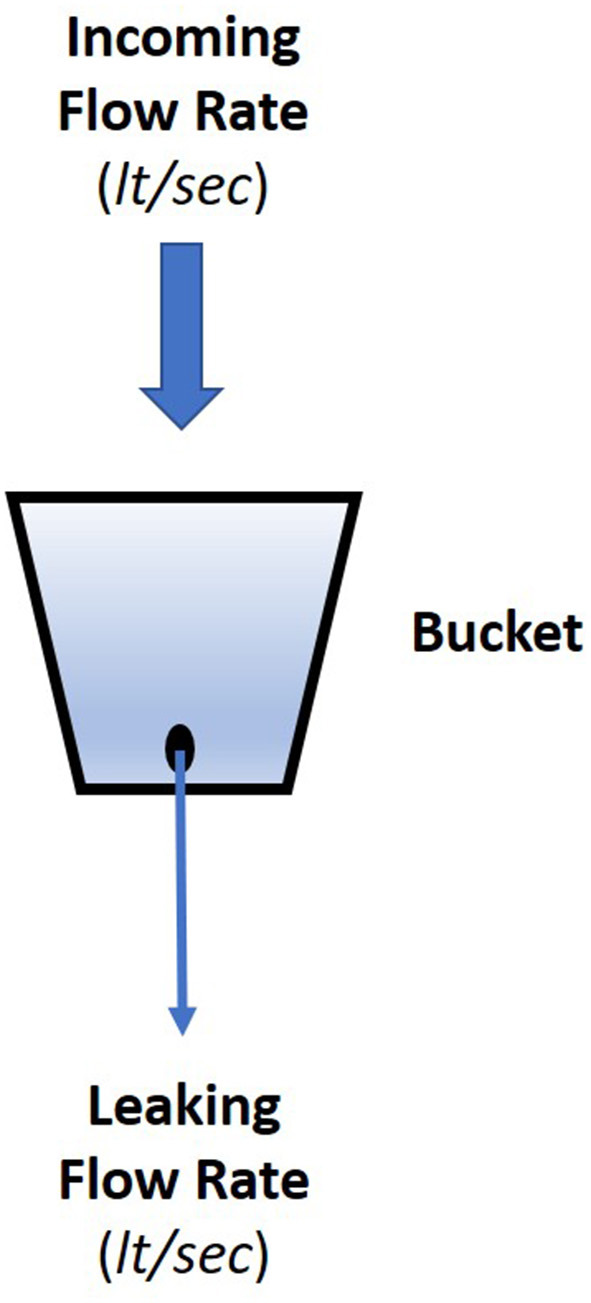
Leaky bucket approach.

**Figure 2:**
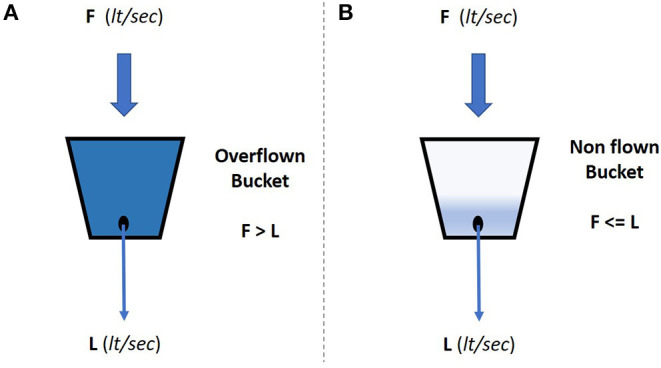
The leaky bucket states: **(A)** overflown, and **(B)** non-overflown.

Visualization of the leaky bucket approach is provided in Figure 1, where the associated flow rates of the water—incoming and leaking—are marked as F and L, respectively. The absolute values of the flow rates define the state of the bucket, which may take one of the two states: (i) the bucket state is defined as *overflown* when the incoming flow rate is higher than the leaking one, i.e., F > L, and (ii) the bucket state is defined as *non-flown* when the leaking rate is higher than the incoming, i.e., F < L. The two states are graphically depicted in Figure 2.

Based on the definition of the leaky bucket, the bucket itself may be viewed in two different ways. In particular, in the first way the bucket is viewed as the analog of an event counter, while in the second way the bucket is viewed as the analog of an event queue. It should be noted that for the purposes of the current manuscript the second view is adopted, i.e., the bucket is viewed as a queue of discrete events-.

**Figure 3:**
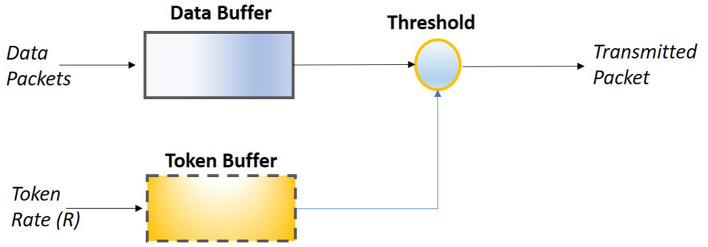
Token driven leaky bucket control of data packet transmission.

Consideration of the leaky bucket as event queue has given rise to another algorithmic that is used in data package flow control and adopts the use of a token rate counter as well. The token driven leaky bucket approach is depicted in Figure 3, where it is observed the concurrent operation of two buffer components—the data and the token buffer-. The first of the buffers, i.e., the data buffer, receives and stores data packets, while the second one, i.e., the token buffer, receives and stores a sequence of tokens that arrive with a rate *R*. The number of tokens stored in the buffer are compared to a predefined threshold value, and when this population of token is equal to exceeds the threshold then a package from the data buffer is transmitted. At this point it should be noted, that the data buffer implements a first-in-first-out queue (FIFO queue) and therefore every time the token threshold is satisfied then the “oldest” packet is transmitted. In addition, the data buffer has limited storage size, causing the buffer to overflown when the package arrival rate is higher than the incoming token rate. In case, the data buffer is overflown then new incoming packets are not stored and eventually are lost. Overall, this approach allows the automated control of the packet transmission via two parameters: the token rate and the threshold value.

Of interest in the current manuscript is the token driven approach. The reason for adopting this approach is that from a broad point of view the packets are the analog of load demand imposed by an electric appliance, while the tokens are the analog of decision factors.

## Fuzzy Leaky Bucket Elastic Load Management System

### Problem Statement

Load management from the demand side refers to scheduling of electric consumption in a way that is optimal for the consumer. Elastic load is comprised of consumption tasks that may be canceled or deferred at a later time. Every task is associated with the operation of an electric appliance. In the current work, the assumption made is that the consumer appliances share intelligent capabilities and they are able to communicate with a smart meter that makes the electricity energy purchases. Furthermore, it is assumed that the smart meter connects to the smart grid and is able to receive information from the grid. In addition, the electricity prices vary dynamically and are announced every 1 h, while they stay valid for the forthcoming hour. Regarding the elastic loads, every intelligent appliance is able to know *a priori* the time it is required to operate for consuming a specific amount of electricity.

Constraints in the consumption are imposed by the physical capacity of the delivery lines. For instance, the lines that deliver the electrical energy have limited capacity *C*. Given that the inelastic load, denoted as *I*, must be satisfied then the maximum elastic that can be scheduled is

$$\begin{array}{l} \text{ME}\left(\text{t}\right)=\text{C}-\text{I}\left(\text{t}\right) \end{array}$$

where *t* stands for time in the form of the hour of the day, i.e., *t* = 1, …, 24. The minimum value of the elastic load is equal to 0 that implies that no elastic load is scheduled.

### Fuzzy Leaky Bucket System

**Figure 4:**
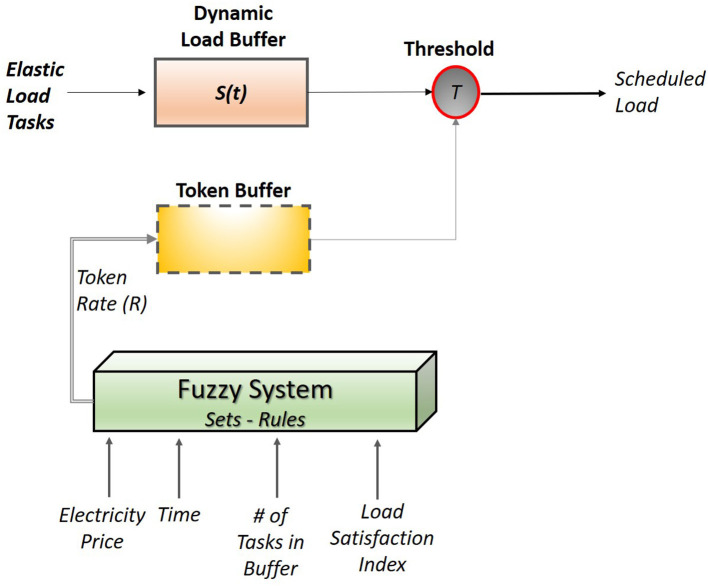
Fuzzy leaky bucket system architecture for elastic load management.

In this section the fuzzy system that implements a leaky bucket approach is presented. The proposed fuzzy leaky bucket system follows a token driven approach; therefore, its architecture, which is depicted in Figure 4, contains two buffer components. In particular, two buffers are: (i) the dynamic load buffer that receives and stores the elastic load tasks, and (ii) the token buffer that receives and stores the tokens.

The aim of the dynamic load buffer is to secure that the amount of scheduled elastic load will never exceed the maximum value ME(*t*). To that end, the storage capacity is considered dynamic, with the storage capacity to be taken equal to ME(*t*) at every hour. By setting the size buffer equal to ME(t), the leaky bucket may schedule tasks whose aggregated load does not exceed ME(t). Thus, the system secures that this limit will be definitely satisfied given that excessive tasks are not stored in the load buffer (so the buffer is identified as overflown).

The token buffer receives the tokens and compares with the threshold T as shown in Figure 4. When the number of tokens in the buffer are equal or exceed T, then the token buffer is emptied and the “oldest” task in the load buffer is released. The rate of token arrival in the buffer, denoted as R, is controlled by a fuzzy system. In particular, R is considered as a fuzzy variable whose value is determined by the fuzzy system at the beginning of every hour.

The fuzzy system applied for token control implements a fuzzy inference mechanism comprised of four inputs, and one output. As presented in Figure 4, the four inputs are:

- The electricity price: this is the value of price announced by the market operator for the hour *t*.
- Time: this represent the current time in the form of hours.
- Number of tasks in buffer: this number is equal to the number of the elastic tasks that have made it into the buffer.
- Load satisfaction index: this index exhibits the utilization of elastic load since the beginning of the day, and it is equal to the ratio
  $$\begin{array}{l} \text{SI}=\frac{\#\text{of tasks scheduled since the beginning of the day}}{\#\text{ of elastic tasks of the day}} \end{array}$$

while the output of the system is:

- The token rate *R*: the number of tokens arriving in the buffer in a single minute.

The goal of the fuzzy system is to determine the token rate at the beginning of each hour in order to implicitly determine the number of scheduled tasks for this specific time. The set of four inputs is selected in such a way that mimics the human way of decision making. In particular, the four inputs were selected on the following rationale:

- The *electricity price* is selected to determine whether it is economically beneficial to schedule a high or a low number of tasks.
- The *time* is used to determined how much is the remaining time until the end of the day. With this variable time constraints are also part of the decision making. It should be mentioned that the tasks are considered on a day by day basis: therefore deferring of a task may happen only within the same day.
- The *number of tasks in the buffer* denotes the resolution of the load. This number shows whether there are many small tasks or a few but large tasks. This information is essential given that the system no matter the size of the load a task is released when the tokens reach the threshold value.
- The *load satisfaction index* quantifies the degree of elastic load satisfaction. In other words, how much of the elastic load for that specific day has been already scheduled. This input is essential in management since it implicitly denotes the remaining elastic load to be scheduled. The goal of every consumer is to fully satisfy his/her demand and this index will express the risk taken to defer some of the load for later scheduling.
- The output variable of token rate will control the scheduling of the tasks. If the above inputs indicate a high token rate then a high number of tasks will be scheduled. If the token rate is low, then a low number of tasks is scheduled. The *R* value is valid for an hour.

**Figure 5:**
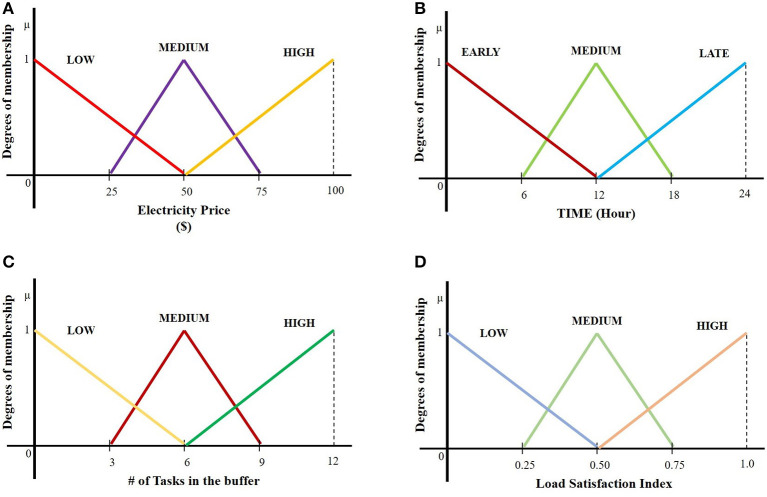
Fuzzy set representation of the input variable: **(A)** “electricity price,” **(B)** “time,” **(C)** “# of tasks in the buffer,” and **(D)** “load satisfaction index”.

The above values are recognized in the current manuscript as fuzzy variables and therefore they are represented by a group of fuzzy values expressed in the form of linguistic terms. Each linguistic term is modeled by a fuzzy set, with the population of fuzzy sets to span the range of values of the variable. The fuzzy sets used for the four input and the single output variables are provided in Figure 5, respectively, where the fuzzy sets are modeled in the form of triangular membership functions, which is a convenient choice.

**Figure 6:**
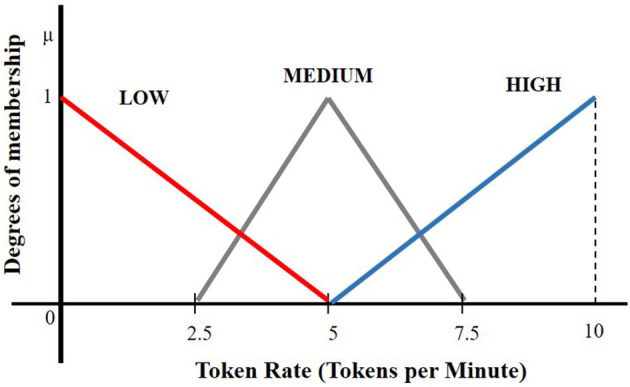
Fuzzy set representation of the output variable “Token rate R”.

The ranges of the values (universe of discourse in fuzzy parlance) as shown in Figures 5, 6 are the following:

➢ 0–100 ($) for electricity price,
➢ 1–24 (h) for time,
➢ 0–12 for # of tasks in the buffer (assuming that every consumer can have max 12 tasks),
➢ 0–1 for load satisfaction index,
➢ 0–10 (tokens/min) for token rate.

The above ranges were selected either based on assumptions, as in # of task in the buffer, or based on design, as in the token rate. The range of electricity prices was selected by observing the prices in one of the major US market operators.

Having defined the fuzzy sets for the input and output variables, then a fuzzy inference engine is developed that associates the inputs to the output. The association is implemented in the form of IF/THEN fuzzy rules given that the rules associate fuzzy sets and take the form:

IF “Conditions”, THEN “Output”

where the conditions consist from one up to four conditions (i.e., four input values). The fuzzy conditions in the left-hand side of the rules consist of fuzzy relations of the form “X is A” with A being a fuzzy set. The conditions are connected with the operator AND, which analytically expresses the max operation among the membership functions of the conditions. Thus, the LHS of the rules are formulated as:

*IF “Condition 1” AND “Condition 2” AND “Condition 3” AND “Condition 4”*,

whose evaluation provides a number that coincides with the maximum degrees of membership value among the conditions. Furthermore, evaluation of the rules is performed with the implication operator *Mamdani Min* (Tsoukalas and Uhrig, 1997) whose analytical formula is given below:

$$\begin{array}{l} \varphi \left(\text{x},\text{ y}\right)\;=\;\mu \left(\text{x}\right)\cap \mu \left(\text{y}\right) \end{array}$$

where the operator retains the minimum of the two membership values.

**Figure 7:**
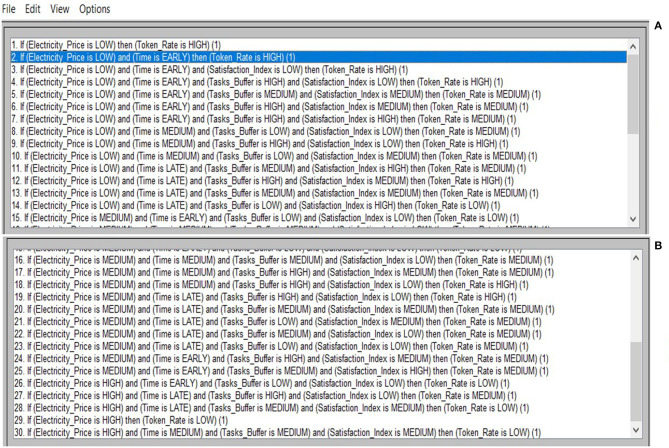
Fuzzy rule based for implementing leaky bucket approach: **(A)** Rules 1–15 and, **(B)** Rules 16–30.

**Figure 8:**
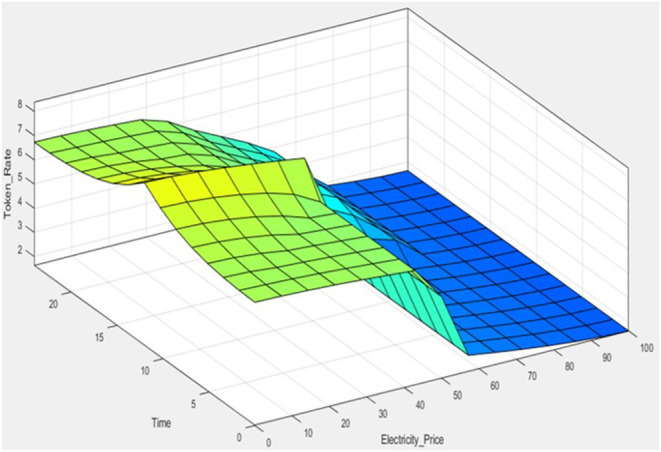
Visualization of decision surface of the fuzzy system.

At this point it should be mentioned that the rule base of the fuzzy leaky bucket system is populated with a set of 30 fuzzy rules. In particular, the encoded rules in the base, which has been developed in the programming environment of the Matlab software (Sivanandam et al., 2007), are given in Figure 7. Given that the output of the fuzzy inference is a fuzzy set then a defuzzification method is applied to obtain a crisp value, and more particularly the Center of Area (CoA) method, whose analytical formula is given by:

$$\begin{array}{l} \text{C}\text{o}\text{A}=\frac{\int_{{\text{x}}_{\min}}^{{\text{x}}_{\max}}\mu \left(x\right)\cdot x\cdot dx}{\int_{{\text{x}}_{\min}}^{{\text{x}}_{\max}}\mu \left(x\right)\cdot dx} \end{array}$$

where *x* is the linguistic (fuzzy) variable and [*x*_*min*_, *x*_*max*_] is the range of the linguistic variable. CoA is selected since it provides the best compromise among multiple output linguistic terms. The decision surface taken with CoA for the developed 30 rule fuzzy system in depicted in Figure 8.

The output of the fuzzy system provides the token rate in the form of tokens/minute. The tokens accumulated in the token rate are continuously compared to a predetermined threshold that in this case has been set equal to 50 tokens:

$$\begin{array}{l} \text{T}=50\;\text{t}\text{o}\text{k}\text{e}\text{n}\text{s}. \end{array}$$

Hence, the above threshold T indicates that the arrival of 50 tokens activates the release and subsequent scheduling of a task from the load buffer, while the token buffer will empty and wait for the new tokens.

## Testing Results On Elastic Load Management

### Test Setup

The goal of management systems is to make optimal decision that will return the highest benefit to the consumer. Regarding elastic load the decisions refer to either canceling or postponing the consumption task. In this manuscript, the presented fuzzy leaky bucket system is applied to a set of real-world datasets taken from a household in France that is publicly available at UCI Repository (2019). The datasets contain the daily consumption pattern in kilowatts (kW) of a single residential consumer as measured from 2006 to 2010. The dataset contains measurement of the resident energy consumption on a minute basis and more details can be found in UCI Repository (2019). In this manuscript, the testing dataset are taken from the year 2007 and more specifically days were sampled from April to December of that year, i.e., 2007. It should be noted that the initial consumption data do not encompass the amount of elastic and inelastic load.

**Figure 9:**
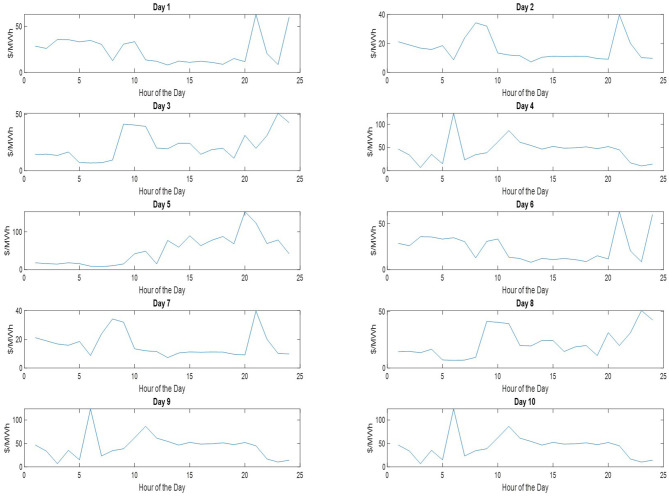
Hourly electricity price signals utilized for computing the daily consumption of the 10 tested days.

In order to separate the overall consumption into elastic and inelastic, we created a randomizer that (randomly) selects the hourly inelastic load. The assumption made in creating the randomizer is that the inelastic load at any time lies between 40 and 70% of the overall consumption. Furthermore, to separate the elastic load into tasks, we developed another randomizer that splits the elastic load amount into one or more tasks: this happens by randomly select a number between 1 and 15 (max number of tasks) and then randomly assign at each task an amount of load under the constraint:

$$\begin{array}{l} Elastic\left(h\right)=\overset{N}{\underset{n=1}{\sum }}\left({\text{Load\_task}}_{\text{n}}\right) \end{array}$$

where *h* stands for the hour of the day and *N* is the number of tasks determined by the randomizer. Regarding the capacity of the consumer, which is denoted as *C*, is taken to be equal to 280 kW (arbitrary selection). In addition, real world electricity prices taken of the same period, i.e., April 2–April 11, are utilized to compute the cost of the electricity consumption. The respective price signals are depicted in Figure 9.

Lastly, the fuzzy leaky bucket is benchmarked against the case that no leaky bucket is adopted and the load is consumed as initially scheduled (i.e., no curtailment of elastic load and no separation between inelastic and elastic loads). Results are recorded and presented with respect to final daily cost of consumption.

### Results

#### Detailed Cases

**Figure 10:**
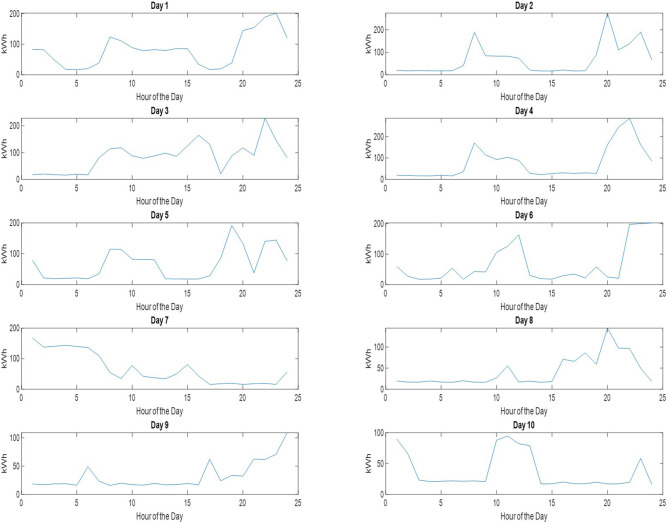
Set of 10 test electricity consumption patterns from the period April 2–April 11 (day 1–10).

In this section the presented elastic consumption management system is applied to the test set comprised of 10 residential consumption patterns that are depicted in Figure 10. Each of the consumption pattern is divided into two different parts, i.e., elastic and inelastic, by using the randomizer that was discussed in the previous section.

In the current work, management results are recorded in the form of daily consumption costs in US dollars. It should be noted that because of the use of randomizer to determine the number and the size of consumption tasks, it is expected that multiple runs on the same dataset will provide different results. To overcome this hurdle in this work, every case is run 13 times and the main statistics of the results are obtained: mean and standard deviation. Notably, the benchmarking method, i.e., scheduling of the initial load without use of leaky bucket denoted as “full scheduling method” in Table 1, makes use of no randomizer and therefore provides a constant cost value independent of the number of runs. It should be noted that the “full scheduling method” assumes no real time decision making and coincides with the demand response methods which determine the consumption schedule in an offline manner.

**Table 1:** Obtained results in the form of daily consumption cost for the tested methods, and percentage of cases where the fuzzy leaky bucket provided higher cost than the full scheduling method.

	**Costs in US dollars ($)**		
	**Fuzzy leaky bucket**		**Full scheduling method**
**Case**	**Mean**	**% of runs above the full scheduling method**	
Day 1	45.82	30%	47.47
Day 2	32.91	23%	34.66
Day 3	47.91	30%	54.16
Day 4	65.01	15%	76.90
Day 5	103.95	15%	118.26
Day 6	70.52	30%	71.51
Day 7	24.81	23%	25.45
Day 8	31.32	0%	38.07
Day 9	43.95	0%	62.55
Day 10	19.94	0%	24.75

Table 1 presents the results obtained with the fuzzy leaky bucket and the benchmark method in the 10 test consumption cases. It is clear from the results that the average cost obtained by fuzzy leaky bucket indeed provides is lower in all tested cases as compared to the “fully scheduling method.” The reduction in cost as compared to the benchmark method, is not uniform for all tested cases. It is observed that for some days—days 1, 2, 6, 7—the reduction in cost is around 10%, while for other days the reduction is above 20% and more specifically for days 3, 4, 5, 8, and 10. Lastly, day 9 provides the highest cost reduction which is around 40%.

**Figure 11:**
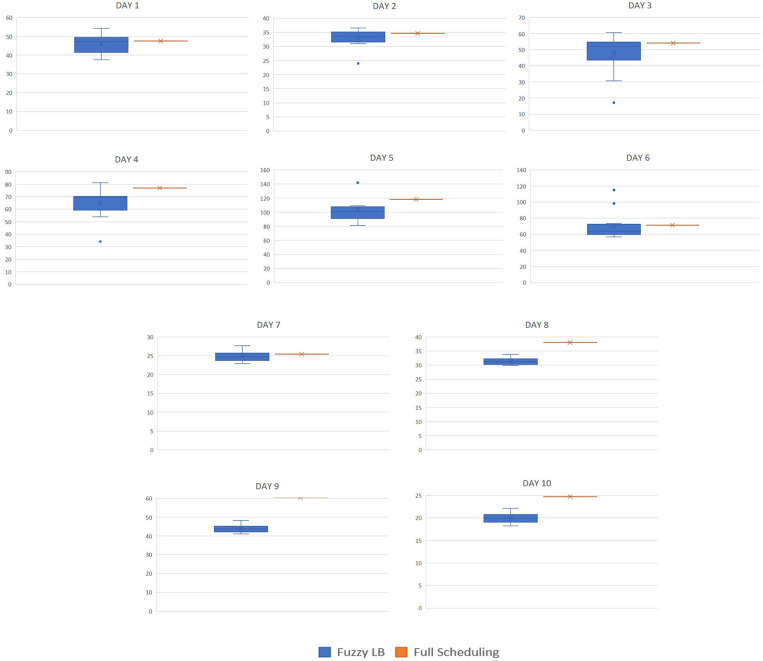
Boxplots for the 13 test runs of each consumption patterns of the period April 2–April 11 (day 1–10).

**Figure 12:**
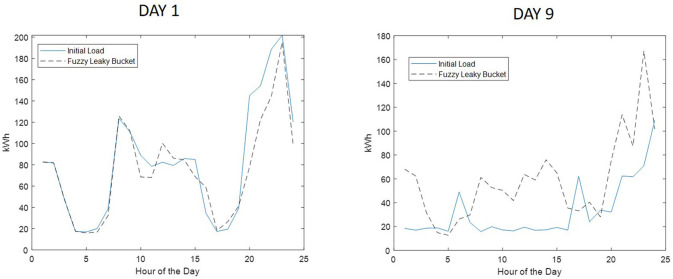
Consumption patterns obtained by fuzzy leaky bucket compared to initial patterns for Day 1 and Day 9 of Table 1.

In addition to costs, Table 1 provides in the third column the percentage of runs that provided consumption cost higher than that of the benchmark. In other words, it shows the number of times that the fuzzy leaky bucket provided a higher cost than the benchmark method. It is observed that the fuzzy leaky bucket is outperformed by the benchmark method only for a small amount of runs. As it is shown in Table 1, the presented method is outperformed at a maximum of 30% for days 1, 3, and 6, while for days 8, 9, and 10, that percentage drops down to 0%. Computing the average percentage per case where the “full scheduling method” provides lower cost turns to be equal to 16.6%. Hence, the fuzzy leaky bucket provides lower cost in the 83.4 of the tested cases. This is also shown in Figure 11 where the box plots of the cost obtained per run per case are given. The boxplots also verify that (i) in the first seven cases there are runs of fuzzy leaky bucket with cost higher than that of full scheduling method, and (ii) in the last three all fuzzy leaky bucket runs provide lower cost than the benchmark method. For visualization purposes, Figure 12 shows the initial consumption pattern against the consumption pattern obtained by the proposed system for Days 1 and 9, where we observe that for Day 1 there was consumption task postponement, while for Day 9 there was essentially curtailment in the elastic consumption (cancelation).

The obtained results confirm the potency of the presented fuzzy system to operate as an autonomous consumption management system. The observations show that the fuzzy leaky bucket is able to reduce the cost in the vast majority of the cases. Furthermore, this reduction came with no human participation at all: the human consumer had not intervention in the process of decision making whether the elastic tasks will be curtailed or postponed. In addition, the proposed method performed efficient even under the cases of high uncertainty. The use of randomizer processes in order to simulate the consumer behavior resulted in seeding of uncertainties within the problem in the form of random number of tasks, random size of tasks. However, the system was able to overcome the inherent uncertainties as it is shown from the fact that it provided the lowest cost in the vast majority of the cases. At last, computationally, the proposed system is inexpensive, while being scalable and easy to update.

#### Further Results

In this section, the proposed fuzzy leaky bucket methodology is further tested for intelligent management of elastic load for load patterns taken from various seasons. Notably, in this section the results are recorded and discussed but no full details are given as in previous section. More specifically, the testing dataset is comprised of 23 residential consumption patterns of the following days of the year 2007 (UCI Repository, 2019): May 1–5, June 11–14, July 16–18, August 23–25, September 12–13, October 2–3, November 16–17, and December 11–12.

**Figure 13:**
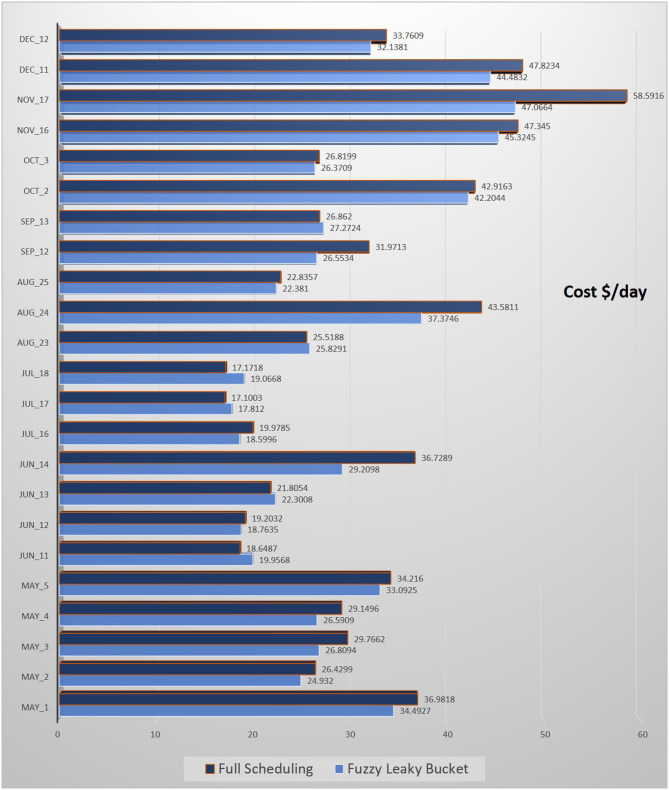
Results obtained in the form of $/day for Fuzzy Leaky Bucket and the benchmark method of full scheduling for a set of 23 days.

The testing of the fuzzy leaky bucket is performed as before: we run the fuzzy leaky bucket for 12 different scenarios and then we compute the consumption cost provided by each scenario. The average cost computed by the 12 scenarios for each of 23 test days is provided in Figure 13, where the cost are computed as $/day). Furthermore, the consumption cost as taken with the full scheduling method (i.e., no fuzzy leaky bucket is used) is also computed and shown in Figure 13 as well.

Results exhibit that the use of fuzzy leaky bucket reduces the consumption cost in the vast majority of the scenarios. More particularly, in 17 out of 23 scenarios the fuzzy leaky bucket average cost is lower than the one obtained with the full scheduling method. Therefore, this implies that the utilization of fuzzy leaky bucket is beneficial to the user in the long run—it may not always provide lower cost but it reduces the cost in the majority of the cases. In the test set in this section, the fuzzy leaky bucket provided lower cost in the 74% of the cases (and higher in the 36% of the cases) as compared to the benchmark method. Therefore, the proposed method clearly outperforms the full scheduling method in the vast majority of the tested cases. The specific cost values obtained by each method on each tested day are also provided in Figure 13.

Overall, the proposed methodology provides a specific set of advantages. The most important of them encompasses the automated scheduling of elastic load tasks at any time of the day. The system required no human intervention at any stage of the testing. Furthermore, the proposed system—in fact that specific implementation of the system—provided lower consumption cost in the vast majority of the cases as compared to full scheduling method. In the cases that the system was beat by the full scheduling method were cases where the randomizer provided high load elastic load thus making the rescheduling less flexible; however, even in those cases the proposed fuzzy leaky bucket provided costs close to the full scheduling. By inspecting the obtained results on all the 33 tested cases, it is concluded that the fuzzy leaky bucket is able to provide lower cost in the long run as compared to a full scheduling method (i.e., that can be considered as a DR method that performs offline scheduling). In sum, the proposed fuzzy leaky bucket system managed to lower the cost in the 84% of the cases examined in the previous section (detailed cases) and in 74% of the cases examined in the current section; putting together all those cases, it is concluded that the presented system outperformed the offline scheduling in the 79% of the cases.

## Conclusion

In this paper a new fuzzy system that implements a leaky bucket management approach was presented and applied to management of elastic load in electricity consumption patterns. The system proposes a new approach by adding a fuzzy inference system in controlling the token rate of the token buffer as implemented in the control approach of leaky bucket. The fuzzy system gets a set of four inputs that are used to mimic the human way of making decisions regarding electricity consumption. Essential part of the proposed system is the fuzzy rule base comprised of 30 rules that associates the inputs to the output. The goal of the systems is to control the token rate in order to control the release of the consumption tasks that are accumulated in the task buffer. In addition, the leaky bucket secures also the safe operation of the system by allowing the task to vary in size and remain within the safety limits. Therefore, the limited size of task buffer allows for rejection of excess tasks ensuring that the capacity of the system won't be exceeded.

The proposed system has been tested in a set of 33 real world consumption patterns. Simulations of consumption behavior were done with the aid of randomizer processes that provided determined in a stochastic behavior the amount of elastic consumption, the number of elastic tasks and their size. Obtained results support the belief that the fuzzy leaky bucket provides lower consumption cost in the vast majority of the test cases-−79% of the cases—compared to the case that it is not used. Furthermore, the proposed system is fully autonomous and requires no human intervention at any stage, while being computationally inexpensive.

As the power grid will become smarter and more information will flow in it, the benefits of using fuzzy leaky bucket for management of elastic load will become more apparent. An advantage of the presented approach is the use of fuzzy rules: their scalability will allow fuzzy leaky bucket system to adapt to new and evolving informational environments.

## Data Availability Statement

Publicly available datasets were analyzed in this study. This data can be found here: https://archive.ics.uci.edu/ml/datasets/Individual+household+electric+power+consumption.

## Author Contributions

The author confirms being the sole contributor of this work and has approved it for publication.

### Conflict of Interest

The author declares that the research was conducted in the absence of any commercial or financial relationships that could be construed as a potential conflict of interest.
